# Supplementary material for: Correlation of national and healthcare workers COVID-19 infection data; implications for large-scale viral testing programs
Source: PLoS One. 2021 Apr 23;16(4):e0250699. doi: 10.1371/journal.pone.0250699 (PMC8064524; doi:10.1371/journal.pone.0250699)
Supplement: S1 File — (PDF) [file pone.0250699.s001.pdf]

S1 File

Correlation of National and Healthcare Workers COVID-19 Infection Data; Implications for Large-scale Viral Testing Programs

Dan Wu,[1] Pól Mac Aonghusa,[2] Donal F. O'Shea*[1]

1 Department of Chemistry, Royal College of Surgeons in Ireland, Dublin 2, Ireland

2 IBM Research – Ireland, Damastown Industrial Estate, Mulhuddart, Dublin 15, Ireland

Legends for Supporting Movies

SI Movie 1. Fitted logistic cures with prediction intervals for Ireland

SI Movie 2. Fitted logistic cures with prediction intervals for South Korea

SI Movie 3. Fitted logistic cures with prediction intervals for Germany

SI Movie 4. Fitted logistic cures with prediction intervals for UK

SI Movie 5. Fitted logistic cures with prediction intervals for Iceland

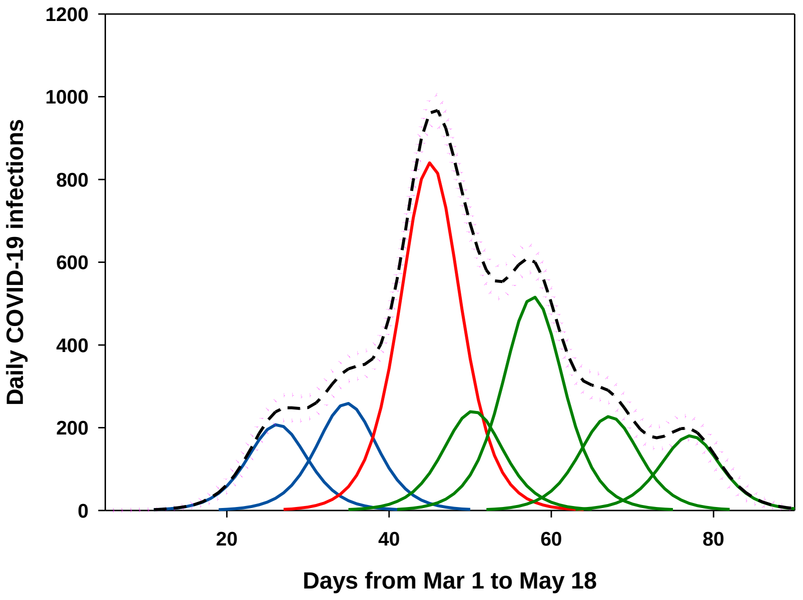

Figure S1. Fitted logistic curves for Ireland showing 95% confidence intervals (dotted purple trace)

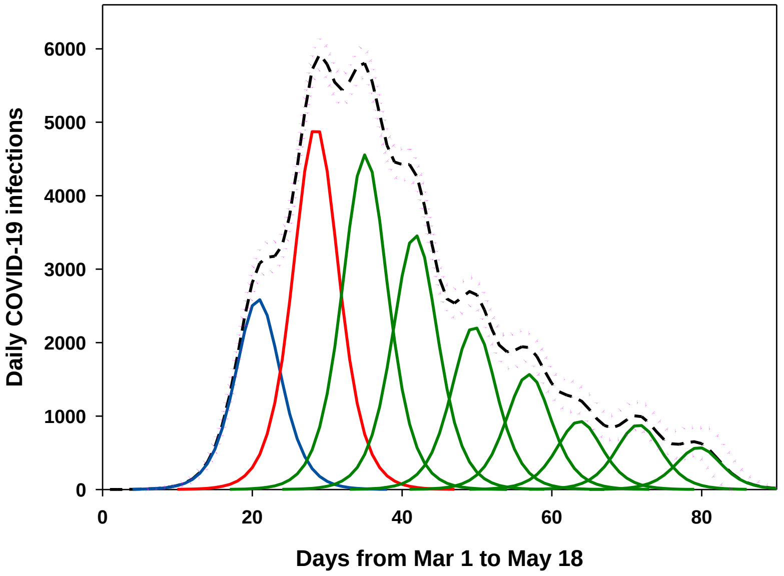

Figure S2. Fitted logistic curves for Germany showing 95% confidence intervals (dotted purple trace)

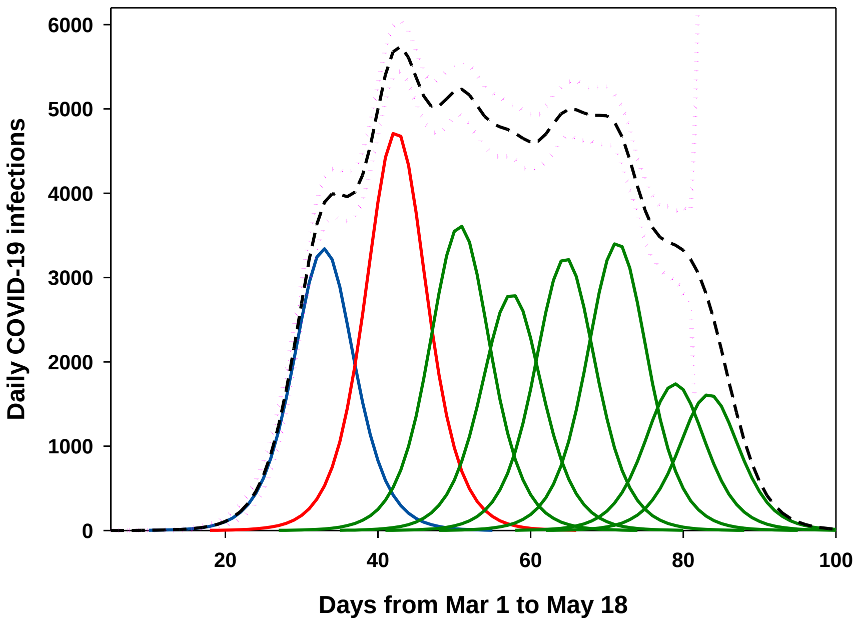

Figure S3. Fitted logistic curves for UK showing 95% confidence intervals (dotted purple trace).

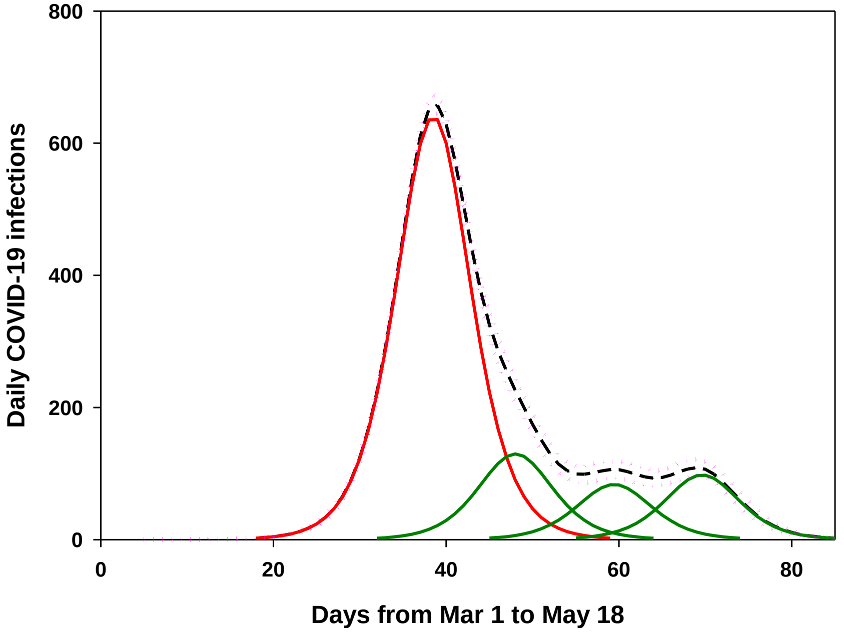

Figure S4. Fitted logistic curves for South Korea showing 95% confidence intervals (dotted purple trace).

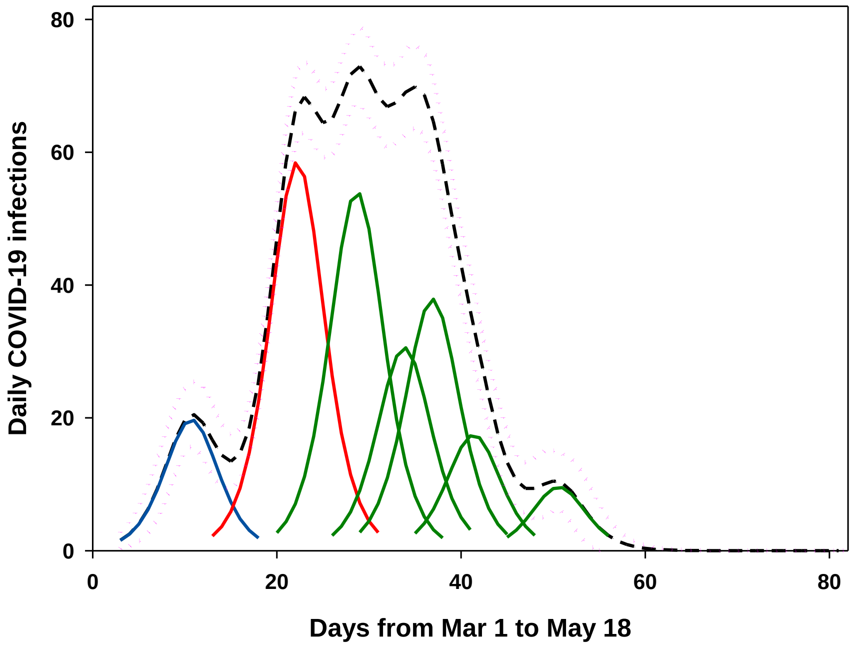

Figure S5. Fitted logistic curves for Iceland showing 95% confidence intervals (dotted purple trace).

Table S1. Data for Spearman test of set and subset

X values	Y values	X_{Ra}	X_{Ra} - M_x	Y_{Ra}	Y_{Ra} - M_y	*Sum Diffs*
185.4	40.4	2.00	-25.00	1.00	-26.00	650.00
213.6	47.4	11.50	-15.50	2.00	-25.00	387.50
250.2	51.8	14.00	-13.00	3.00	-24.00	312.00
262.4	59	16.00	-11.00	4.00	-23.00	253.00
259.6	65.2	15.00	-12.00	5.00	-22.00	264.00
271	75.4	17.00	-10.00	6.00	-21.00	210.00
276.4	79.2	18.00	-9.00	7.00	-20.00	180.00
288.6	88.4	20.00	-7.00	14.00	-13.00	91.00
308.4	81.2	22.00	-5.00	8.00	-19.00	95.00
353.4	83.2	26.00	-1.00	12.00	-15.00	15.00
385.4	82.2	28.00	1.00	10.50	-16.50	-16.50
462.4	89.4	31.00	4.00	15.00	-12.00	-48.00
466	104	32.00	5.00	20.00	-7.00	-35.00
486.2	136.2	33.00	6.00	29.00	2.00	12.00
525.6	157.2	35.00	8.00	33.00	6.00	48.00
574.2	175.6	38.00	11.00	39.00	12.00	132.00
581.4	184.8	40.00	13.00	43.00	16.00	208.00
700.8	184.2	46.00	19.00	41.00	14.00	266.00
789	188.4	48.00	21.00	45.00	18.00	378.00
838.8	184.6	49.00	22.00	42.00	15.00	330.00
928.4	189.8	53.00	26.00	46.00	19.00	494.00
903.2	207	52.00	25.00	48.00	21.00	525.00
852.4	216.8	51.00	24.00	51.00	24.00	576.00
845.2	216.2	50.00	23.00	50.00	23.00	529.00
785.8	227.4	47.00	20.00	53.00	26.00	520.00
610.4	218	43.00	16.00	52.00	25.00	400.00
585.8	209.2	41.00	14.00	49.00	22.00	308.00
553	194.4	36.00	9.00	47.00	20.00	180.00
577.2	185	39.00	12.00	44.00	17.00	204.00
572.4	167.6	37.00	10.00	36.00	9.00	90.00
608.8	176.8	42.00	15.00	40.00	13.00	195.00
654	162.2	45.00	18.00	34.00	7.00	126.00
623	173.8	44.00	17.00	38.00	11.00	187.00
493.8	171	34.00	7.00	37.00	10.00	70.00
460.4	167.4	30.00	3.00	35.00	8.00	24.00
446.4	155.2	29.00	2.00	32.00	5.00	10.00
354.8	153.8	27.00	0.00	31.00	4.00	0.00
343.6	130.8	24.00	-3.00	26.00	-1.00	3.00
350.2	128.6	25.00	-2.00	25.00	-2.00	4.00
318.4	121.8	23.00	-4.00	23.00	-4.00	16.00
288.4	110.6	19.00	-8.00	22.00	-5.00	40.00
297.6	105	21.00	-6.00	21.00	-6.00	36.00
248.8	103.6	13.00	-14.00	19.00	-8.00	112.00
213	91.6	10.00	-17.00	17.00	-10.00	170.00
202.6	95.6	7.00	-20.00	18.00	-9.00	180.00

208.4	88.2	8.50	-18.50	13.00	-14.00	259.00
192	81.6	6.00	-21.00	9.00	-18.00	378.00
186.8	82.2	3.00	-24.00	10.50	-16.50	396.00
173.6	90.8	1.00	-26.00	16.00	-11.00	286.00
213.6	131.2	11.50	-15.50	27.00	0.00	0.00
208.4	139.2	8.50	-18.50	30.00	3.00	-55.50
191.6	132	5.00	-22.00	28.00	1.00	-22.00
190.8	123.6	4.00	-23.00	24.00	-3.00	69.00

Covariance = 10041.5 / 52 = 193.11
R = 193.11 / (15.44 * 15.44) = 0.81

rs = 0.80972, p (2-tailed) = 0.

Table S2. Data for Spearman test of set-subset and subset.

X values	Y values	X_{Ra}	X_{Ra} - M_x	Y_{Ra}	Y_{Ra} - M_y	*Sum Diffs*
145	40.4	11.00	-16.00	1.00	-26.00	416.00
166.2	47.4	13.00	-14.00	2.00	-25.00	350.00
198.4	51.8	20.00	-7.00	3.00	-24.00	168.00
203.4	59	23.00	-4.00	4.00	-23.00	92.00
194.4	65.2	16.00	-11.00	5.00	-22.00	242.00
195.6	75.4	17.00	-10.00	6.00	-21.00	210.00
197.2	79.2	19.00	-8.00	7.00	-20.00	160.00
200.2	88.4	21.00	-6.00	14.00	-13.00	78.00
227.2	81.2	26.00	-1.00	8.00	-19.00	19.00
270.2	83.2	27.00	0.00	12.00	-15.00	0.00
303.2	82.2	30.00	3.00	10.50	-16.50	-49.50
373	89.4	36.00	9.00	15.00	-12.00	-108.00
362	104	34.00	7.00	20.00	-7.00	-49.00
350	136.2	32.00	5.00	29.00	2.00	10.00
368.4	157.2	35.00	8.00	33.00	6.00	48.00
398.6	175.6	41.00	14.00	39.00	12.00	168.00
396.6	184.8	40.00	13.00	43.00	16.00	208.00
516.6	184.2	46.00	19.00	41.00	14.00	266.00
600.6	188.4	48.00	21.00	45.00	18.00	378.00
654.2	184.6	51.00	24.00	42.00	15.00	360.00
738.6	189.8	53.00	26.00	46.00	19.00	494.00
696.2	207	52.00	25.00	48.00	21.00	525.00
635.6	216.8	50.00	23.00	51.00	24.00	552.00
629	216.2	49.00	22.00	50.00	23.00	506.00
558.4	227.4	47.00	20.00	53.00	26.00	520.00
392.4	218	39.00	12.00	52.00	25.00	300.00
376.6	209.2	37.00	10.00	49.00	22.00	220.00
358.6	194.4	33.00	6.00	47.00	20.00	120.00

392.2	185	38.00	11.00	44.00	17.00	187.00
404.8	167.6	42.00	15.00	36.00	9.00	135.00
432	176.8	43.00	16.00	40.00	13.00	208.00
491.8	162.2	45.00	18.00	34.00	7.00	126.00
449.2	173.8	44.00	17.00	38.00	11.00	187.00
322.8	171	31.00	4.00	37.00	10.00	40.00
293	167.4	29.00	2.00	35.00	8.00	16.00
291.2	155.2	28.00	1.00	32.00	5.00	5.00
201	153.8	22.00	-5.00	31.00	4.00	-20.00
212.8	130.8	24.00	-3.00	26.00	-1.00	3.00
221.6	128.6	25.00	-2.00	25.00	-2.00	4.00
196.6	121.8	18.00	-9.00	23.00	-4.00	36.00
177.8	110.6	14.00	-13.00	22.00	-5.00	65.00
192.6	105	15.00	-12.00	21.00	-6.00	72.00
145.2	103.6	12.00	-15.00	19.00	-8.00	120.00
121.4	91.6	10.00	-17.00	17.00	-10.00	170.00
107	95.6	7.00	-20.00	18.00	-9.00	180.00
120.2	88.2	9.00	-18.00	13.00	-14.00	252.00
110.4	81.6	8.00	-19.00	9.00	-18.00	342.00
104.6	82.2	6.00	-21.00	10.50	-16.50	346.50
82.8	90.8	5.00	-22.00	16.00	-11.00	242.00
82.4	131.2	4.00	-23.00	27.00	0.00	0.00
69.2	139.2	3.00	-24.00	30.00	3.00	-72.00
59.6	132	1.00	-26.00	28.00	1.00	-26.00
67.2	123.6	2.00	-25.00	24.00	-3.00	75.00

Covariance = 8897 / 52 = 171.1
R = 171.1 / (15.44 * 15.44) = 0.717
rs = 0.7174, p (2-tailed) = 0.

Table S3: Data for Spearman test of set and set-subset.

X values	Y values	X_{Ra}	X_{Ra} - M_x	Y_{Ra}	Y_{Ra} - M_y	*Sum Diffs*
185.4	145	2.00	-25.00	11.00	-16.00	400.00
213.6	166.2	11.50	-15.50	13.00	-14.00	217.00
250.2	198.4	14.00	-13.00	20.00	-7.00	91.00
262.4	203.4	16.00	-11.00	23.00	-4.00	44.00
259.6	194.4	15.00	-12.00	16.00	-11.00	132.00
271	195.6	17.00	-10.00	17.00	-10.00	100.00
276.4	197.2	18.00	-9.00	19.00	-8.00	72.00
288.6	200.2	20.00	-7.00	21.00	-6.00	42.00
308.4	227.2	22.00	-5.00	26.00	-1.00	5.00
353.4	270.2	26.00	-1.00	27.00	0.00	0.00

385.4	303.2	28.00	1.00	30.00	3.00	3.00
462.4	373	31.00	4.00	36.00	9.00	36.00
466	362	32.00	5.00	34.00	7.00	35.00
486.2	350	33.00	6.00	32.00	5.00	30.00
525.6	368.4	35.00	8.00	35.00	8.00	64.00
574.2	398.6	38.00	11.00	41.00	14.00	154.00
581.4	396.6	40.00	13.00	40.00	13.00	169.00
700.8	516.6	46.00	19.00	46.00	19.00	361.00
789	600.6	48.00	21.00	48.00	21.00	441.00
838.8	654.2	49.00	22.00	51.00	24.00	528.00
928.4	738.6	53.00	26.00	53.00	26.00	676.00
903.2	696.2	52.00	25.00	52.00	25.00	625.00
852.4	635.6	51.00	24.00	50.00	23.00	552.00
845.2	629	50.00	23.00	49.00	22.00	506.00
785.8	558.4	47.00	20.00	47.00	20.00	400.00
610.4	392.4	43.00	16.00	39.00	12.00	192.00
585.8	376.6	41.00	14.00	37.00	10.00	140.00
553	358.6	36.00	9.00	33.00	6.00	54.00
577.2	392.2	39.00	12.00	38.00	11.00	132.00
572.4	404.8	37.00	10.00	42.00	15.00	150.00
608.8	432	42.00	15.00	43.00	16.00	240.00
654	491.8	45.00	18.00	45.00	18.00	324.00
623	449.2	44.00	17.00	44.00	17.00	289.00
493.8	322.8	34.00	7.00	31.00	4.00	28.00
460.4	293	30.00	3.00	29.00	2.00	6.00
446.4	291.2	29.00	2.00	28.00	1.00	2.00
354.8	201	27.00	0.00	22.00	-5.00	0.00
343.6	212.8	24.00	-3.00	24.00	-3.00	9.00
350.2	221.6	25.00	-2.00	25.00	-2.00	4.00
318.4	196.6	23.00	-4.00	18.00	-9.00	36.00
288.4	177.8	19.00	-8.00	14.00	-13.00	104.00
297.6	192.6	21.00	-6.00	15.00	-12.00	72.00
248.8	145.2	13.00	-14.00	12.00	-15.00	210.00
213	121.4	10.00	-17.00	10.00	-17.00	289.00
202.6	107	7.00	-20.00	7.00	-20.00	400.00
208.4	120.2	8.50	-18.50	9.00	-18.00	333.00
192	110.4	6.00	-21.00	8.00	-19.00	399.00
186.8	104.6	3.00	-24.00	6.00	-21.00	504.00
173.6	82.8	1.00	-26.00	5.00	-22.00	572.00
213.6	82.4	11.50	-15.50	4.00	-23.00	356.50
208.4	69.2	8.50	-18.50	3.00	-24.00	444.00
191.6	59.6	5.00	-22.00	1.00	-26.00	572.00
190.8	67.2	4.00	-23.00	2.00	-25.00	575.00

Covariance = 12119.5 / 52 = 233.07
R = 233.07 / (15.44 * 15.44) = 0.977
rs = 0.97726, p (2-tailed) = 0.

Table S4: Data for Pearson's coefficient test of set and subset

X values	Y values	X - M_x	Y - My	(X - M_x)^2	(Y - M_y)^2	(X - M_x)(Y - M_y)
185.4000	40.4000	-251.611	-93.672	63308.257	8774.387	23568.860
213.6000	47.4000	-223.411	-86.672	49912.618	7511.983	19363.439
250.2000	51.8000	-186.811	-82.272	34898.470	6768.632	15369.285
262.4000	59.0000	-174.611	-75.072	30489.113	5635.760	13108.368
259.6000	65.2000	-177.411	-68.872	31474.777	4743.311	12218.619
271.0000	75.4000	-166.011	-58.672	27559.759	3442.368	9740.166
276.4000	79.2000	-160.611	-54.872	25795.996	3010.903	8813.016
288.6000	88.4000	-148.411	-45.672	22025.920	2085.904	6778.197
308.4000	81.2000	-128.611	-52.872	16540.872	2795.416	6799.899
353.4000	83.2000	-83.611	-50.872	6990.853	2587.930	4253.450
385.4000	82.2000	-51.611	-51.872	2663.728	2690.673	2677.167
462.4000	89.4000	25.389	-44.672	644.585	1995.561	-1134.155
466.0000	104.0000	28.989	-30.072	840.344	904.307	-871.739
486.2000	136.2000	49.189	2.128	2419.526	4.530	104.688
525.6000	157.2000	88.589	23.128	7847.954	534.918	2048.906
574.2000	175.6000	137.189	41.528	18820.734	1724.600	5697.213
581.4000	184.8000	144.389	50.728	20848.091	2573.361	7324.593
700.8000	184.2000	263.789	50.128	69584.467	2512.847	13223.279
789.0000	188.4000	351.989	54.328	123896.030	2951.564	19122.947
838.8000	184.6000	401.789	50.528	161434.143	2553.109	20301.700
928.4000	189.8000	491.389	55.728	241462.834	3105.644	27384.257
903.2000	207.0000	466.189	72.928	217331.885	5318.537	33998.349
852.4000	216.8000	415.389	82.728	172547.755	6843.972	34364.400
845.2000	216.2000	408.189	82.128	166617.998	6745.058	33523.843
785.8000	227.4000	348.789	93.328	121653.543	8710.172	32551.855
610.4000	218.0000	173.389	83.928	30063.634	7043.960	14552.217
585.8000	209.2000	148.789	75.128	22138.071	5644.262	11178.241
553.0000	194.4000	115.989	60.328	13453.374	3639.504	6997.400
577.2000	185.0000	140.189	50.928	19652.866	2593.692	7139.571
572.4000	167.6000	135.389	33.528	18330.094	1124.147	4539.353
608.8000	176.8000	171.789	42.728	29511.350	1825.708	7340.239
654.0000	162.2000	216.989	28.128	47084.087	791.201	6103.523
623.0000	173.8000	185.989	39.728	34591.789	1578.338	7389.014

493.8000	171.0000	56.789	36.928	3224.954	1363.699	2097.109
460.4000	167.4000	23.389	33.328	547.030	1110.776	779.505
446.4000	155.2000	9.389	21.128	88.147	446.405	198.367
354.8000	153.8000	-82.211	19.728	6758.701	389.206	-1621.890
343.6000	130.8000	-93.411	-3.272	8725.675	10.704	305.614
350.2000	128.6000	-86.811	-5.472	7536.205	29.939	475.005
318.4000	121.8000	-118.611	-12.272	14068.645	150.595	1455.562
288.4000	110.6000	-148.611	-23.472	22085.325	550.921	3488.160
297.6000	105.0000	-139.411	-29.072	19435.516	845.164	4052.924
248.8000	103.6000	-188.211	-30.472	35423.501	928.524	5735.119
213.0000	91.6000	-224.011	-42.472	50181.072	1803.845	9514.141
202.6000	95.6000	-234.411	-38.472	54948.667	1480.072	9018.202
208.4000	88.2000	-228.611	-45.872	52263.136	2104.213	10486.789
192.0000	81.6000	-245.011	-52.472	60030.547	2753.279	12856.160
186.8000	82.2000	-250.211	-51.872	62605.705	2690.673	12978.886
173.6000	90.8000	-263.411	-43.272	69385.524	1872.440	11398.255
213.6000	131.2000	-223.411	-2.872	49912.618	8.247	641.570
208.4000	139.2000	-228.611	5.128	52263.136	26.299	-1172.388
191.6000	132.0000	-245.411	-2.072	60226.716	4.292	508.418
190.8000	123.6000	-246.211	-10.472	60620.014	109.656	2578.251
		Mx: 437.011	My: 134.072	Sum: 2542766.353	Sum: 139445.208	Sum: 501343.917

X Values

∑ = 23161.6; Mean = 437.011; ∑(X - Mx)2 = SSx = 2542766.353

Y Values

∑ = 7105.8; Mean = 134.072; ∑(Y - My)2 = SSy = 139445.208

X and Y Combined

N = 53; ∑(X - Mx)(Y - My) = 501343.917

R Calculation; r = ∑((X - My)(Y - Mx)) / √((SSx)(SSy))

r = 501343.917 / √((2542766.353)(139445.208)) = 0.8419

The P-Value is < .00001. The result is significant at p < .01.

Table S5. Data for Pearson's coefficient of set-subset and subset.

X values	Y values	X - M_x	Y - My	(X - $M_x)^2$	(Y - $M_y)^2$	(X - M_x)(Y - M_y)
145.0000	40.4000	-157.940	-93.672	24944.924	8774.387	14794.473
166.2000	47.4000	-136.740	-86.672	18697.724	7511.983	11851.455
198.4000	51.8000	-104.540	-82.272	10928.533	6768.632	8600.652
203.4000	59.0000	-99.540	-75.072	9908.136	5635.760	7472.609
194.4000	65.2000	-108.540	-68.872	11780.850	4743.311	7475.308
195.6000	75.4000	-107.340	-58.672	11521.795	3442.368	6297.798
197.2000	79.2000	-105.740	-54.872	11180.868	3010.903	5802.113
200.2000	88.4000	-102.740	-45.672	10555.430	2085.904	4692.293
227.2000	81.2000	-75.740	-52.872	5736.490	2795.416	4004.482
270.2000	83.2000	-32.740	-50.872	1071.883	2587.930	1665.520
303.2000	82.2000	0.260	-51.872	0.068	2690.673	-13.506
373.0000	89.4000	70.060	-44.672	4908.456	1995.561	-3129.716
362.0000	104.0000	59.060	-30.072	3488.128	904.307	-1776.046
350.0000	136.2000	47.060	2.128	2214.679	4.530	100.159
368.4000	157.2000	65.460	23.128	4285.061	534.918	1513.987
398.6000	175.6000	95.660	41.528	9150.908	1724.600	3972.613
396.6000	184.8000	93.660	50.728	8772.266	2573.361	4751.232
516.6000	184.2000	213.660	50.128	45650.757	2512.847	10710.432
600.6000	188.4000	297.660	54.328	88601.700	2951.564	16171.383
654.2000	184.6000	351.260	50.528	123383.853	2553.109	17748.590
738.6000	189.8000	435.660	55.728	189799.964	3105.644	24278.613
696.2000	207.0000	393.260	72.928	154653.724	5318.537	28679.812
635.6000	216.8000	332.660	82.728	110662.927	6843.972	27520.428
629.0000	216.2000	326.060	82.128	106315.370	6745.058	26778.785
558.4000	227.4000	255.460	93.328	65260.004	8710.172	23841.683
392.4000	218.0000	89.460	83.928	8003.159	7043.960	7508.258
376.6000	209.2000	73.660	75.128	5425.851	5644.262	5533.979
358.6000	194.4000	55.660	60.328	3098.078	3639.504	3357.896
392.2000	185.0000	89.260	50.928	7967.415	2593.692	4545.879
404.8000	167.6000	101.860	33.528	10375.536	1124.147	3415.205
432.0000	176.8000	129.060	42.728	16656.581	1825.708	5514.531
491.8000	162.2000	188.860	28.128	35668.242	791.201	5312.322
449.2000	173.8000	146.260	39.728	21392.098	1578.338	5810.676
322.8000	171.0000	19.860	36.928	394.435	1363.699	733.410
293.0000	167.4000	-9.940	33.328	98.796	1110.776	-331.271
291.2000	155.2000	-11.740	21.128	137.819	446.405	-248.038
201.0000	153.8000	-101.940	19.728	10391.687	389.206	-2011.096
212.8000	130.8000	-90.140	-3.272	8125.152	10.704	294.910
221.6000	128.6000	-81.340	-5.472	6616.134	29.939	445.066
196.6000	121.8000	-106.340	-12.272	11308.115	150.595	1304.968
177.8000	110.6000	-125.140	-23.472	15659.925	550.921	2937.239

192.6000	105.0000	-110.340	-29.072	12174.832	845.164	3207.760
145.2000	103.6000	-157.740	-30.472	24881.789	928.524	4806.594
121.4000	91.6000	-181.540	-42.472	32956.635	1803.845	7710.296
107.0000	95.6000	-195.940	-38.472	38392.336	1480.072	7538.130
120.2000	88.2000	-182.740	-45.872	33393.770	2104.213	8382.577
110.4000	81.6000	-192.540	-52.472	37071.506	2753.279	10102.881
104.6000	82.2000	-198.340	-51.872	39338.606	2690.673	10288.213
82.8000	90.8000	-220.140	-43.272	48461.453	1872.440	9525.815
82.4000	131.2000	-220.540	-2.872	48637.725	8.247	633.323
69.2000	139.2000	-233.740	5.128	54634.211	26.299	-1198.687
59.6000	132.0000	-243.340	-2.072	59214.172	4.292	504.126
67.2000	123.6000	-235.740	-10.472	55573.170	109.656	2468.594
		Mx: 302.940	My: 134.072	Sum: 1679523.727	Sum: 139445.208	Sum: 361898.709

X Values

∑ = 16055.8; Mean = 302.94; ∑(X - Mx)2 = SSx = 1679523.727

Y Values

∑ = 7105.8; Mean = 134.072; ∑(Y - My)2 = SSy = 139445.208

X and Y Combined

N = 53; ∑(X - Mx)(Y - My) = 361898.709

R Calculation

r = ∑((X - My)(Y - Mx)) / √((SSx)(SSy))

r = 361898.709 / √((1679523.727)(139445.208)) = 0.7478

The P-Value is < .00001. The result is significant at p < .01.

Table S6. Data for Pearson's coefficient of set and set-subset

X values	Y values	X - M_x	Y - My	$(X - M_x)^2$	$(Y - M_y)^2$	$(X - M_x)(Y - M_y)$
185.4000	145.0000	-251.611	-157.940	63308.257	24944.924	39739.397
213.6000	166.2000	-223.411	-136.740	49912.618	18697.724	30549.180
250.2000	198.4000	-186.811	-104.540	34898.470	10928.533	19529.185
262.4000	203.4000	-174.611	-99.540	30489.113	9908.136	17380.745
259.6000	194.4000	-177.411	-108.540	31474.777	11780.850	19256.158
271.0000	195.6000	-166.011	-107.340	27559.759	11521.795	17819.593
276.4000	197.2000	-160.611	-105.740	25795.996	11180.868	16982.980
288.6000	200.2000	-148.411	-102.740	22025.920	10555.430	15247.723
308.4000	227.2000	-128.611	-75.740	16540.872	5736.490	9740.973
353.4000	270.2000	-83.611	-32.740	6990.853	1071.883	2737.403
385.4000	303.2000	-51.611	0.260	2663.728	0.068	-13.438
462.4000	373.0000	25.389	70.060	644.585	4908.456	1778.740
466.0000	362.0000	28.989	59.060	840.344	3488.128	1712.082

486.2000	350.0000	49.189	47.060	2419.526	2214.679	2314.838
525.6000	368.4000	88.589	65.460	7847.954	4285.061	5799.048
574.2000	398.6000	137.189	95.660	18820.734	9150.908	13123.521
581.4000	396.6000	144.389	93.660	20848.091	8772.266	13523.498
700.8000	516.6000	263.789	213.660	69584.467	45650.757	56361.189
789.0000	600.6000	351.989	297.660	123896.030	88601.700	104773.083
838.8000	654.2000	401.789	351.260	161434.143	123383.853	141132.443
928.4000	738.6000	491.389	435.660	241462.834	189799.964	214078.577
903.2000	696.2000	466.189	393.260	217331.885	154653.724	183333.536
852.4000	635.6000	415.389	332.660	172547.755	110662.927	138183.355
845.2000	629.0000	408.189	326.060	166617.998	106315.370	133094.155
785.8000	558.4000	348.789	255.460	121653.543	65260.004	89101.688
610.4000	392.4000	173.389	89.460	30063.634	8003.159	15511.417
585.8000	376.6000	148.789	73.660	22138.071	5425.851	10959.830
553.0000	358.6000	115.989	55.660	13453.374	3098.078	6455.974
577.2000	392.2000	140.189	89.260	19652.866	7967.415	12513.294
572.4000	404.8000	135.389	101.860	18330.094	10375.536	13790.742
608.8000	432.0000	171.789	129.060	29511.350	16656.581	22171.112
654.0000	491.8000	216.989	188.860	47084.087	35668.242	40980.564
623.0000	449.2000	185.989	146.260	34591.789	21392.098	27202.774
493.8000	322.8000	56.789	19.860	3224.954	394.435	1127.845
460.4000	293.0000	23.389	-9.940	547.030	98.796	-232.475
446.4000	291.2000	9.389	-11.740	88.147	137.819	-110.220
354.8000	201.0000	-82.211	-101.940	6758.701	10391.687	8380.591
343.6000	212.8000	-93.411	-90.140	8725.675	8125.152	8420.061
350.2000	221.6000	-86.811	-81.340	7536.205	6616.134	7061.200
318.4000	196.6000	-118.611	-106.340	14068.645	11308.115	12613.083
288.4000	177.8000	-148.611	-125.140	22085.325	15659.925	18597.165
297.6000	192.6000	-139.411	-110.340	19435.516	12174.832	15382.593
248.8000	145.2000	-188.211	-157.740	35423.501	24881.789	29688.383
213.0000	121.4000	-224.011	-181.540	50181.072	32956.635	40666.931
202.6000	107.0000	-234.411	-195.940	54948.667	38392.336	45930.466
208.4000	120.2000	-228.611	-182.740	52263.136	33393.770	41776.346
192.0000	110.4000	-245.011	-192.540	60030.547	37071.506	47174.387
186.8000	104.6000	-250.211	-198.340	62605.705	39338.606	49626.819
173.6000	82.8000	-263.411	-220.140	69385.524	48461.453	57987.269
213.6000	82.4000	-223.411	-220.540	49912.618	48637.725	49271.048
208.4000	69.2000	-228.611	-233.740	52263.136	54634.211	53435.524
191.6000	59.6000	-245.411	-243.340	60226.716	59214.172	59718.298
190.8000	67.2000	-246.211	-235.740	60620.014	55573.170	58041.764
		Mx: 437.011	My: 302.940	Sum: 2542766.353	Sum: 1679523.727	Sum: 2041422.436

X Values
∑ = 23161.6; Mean = 437.011; ∑(X - Mx)2 = SSx = 2542766.353
Y Values; ∑ = 16055.8; Mean = 302.94; ∑(Y - My)2 = SSy = 1679523.727
X and Y Combined
N = 53; ∑(X - Mx)(Y - My) = 2041422.436
R Calculation
r = ∑((X - My)(Y - Mx)) / √((SSx)(SSy))
r = 2041422.436 / √((2542766.353)(1679523.727)) = 0.9878
The P-Value is < .00001. The result is significant at p < .01.
