# Supplementary figures and images for: Correlation of national and healthcare workers COVID-19 infection data; implications for large-scale viral testing programs
Source: PLoS One. 2021 Apr 23;16(4):e0250699. doi: 10.1371/journal.pone.0250699 (PMC8064524; doi:10.1371/journal.pone.0250699)

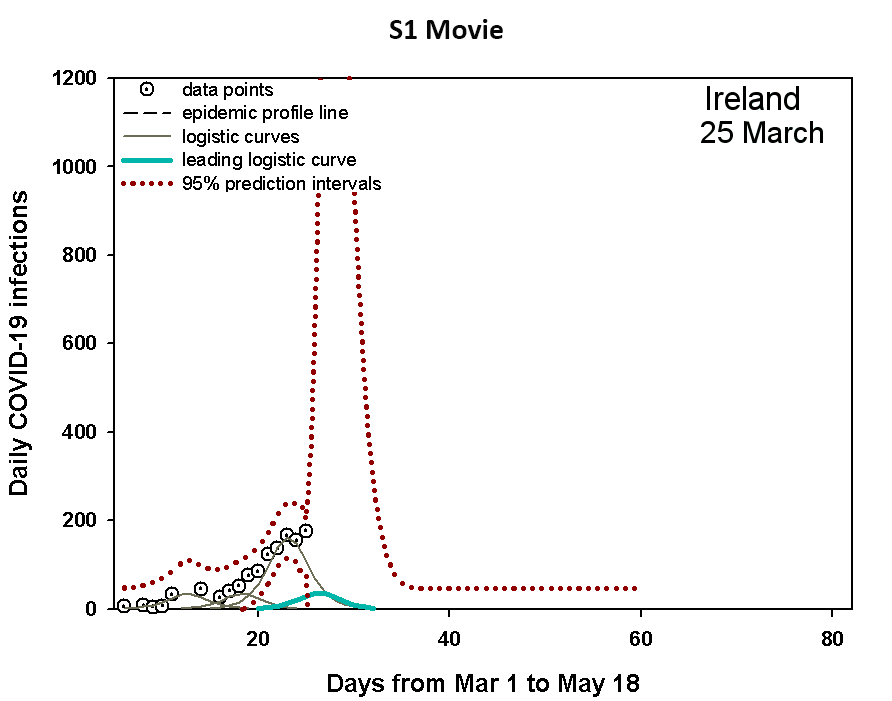

Supplement: S1 Movie — (GIF) [file pone.0250699.s002.gif]

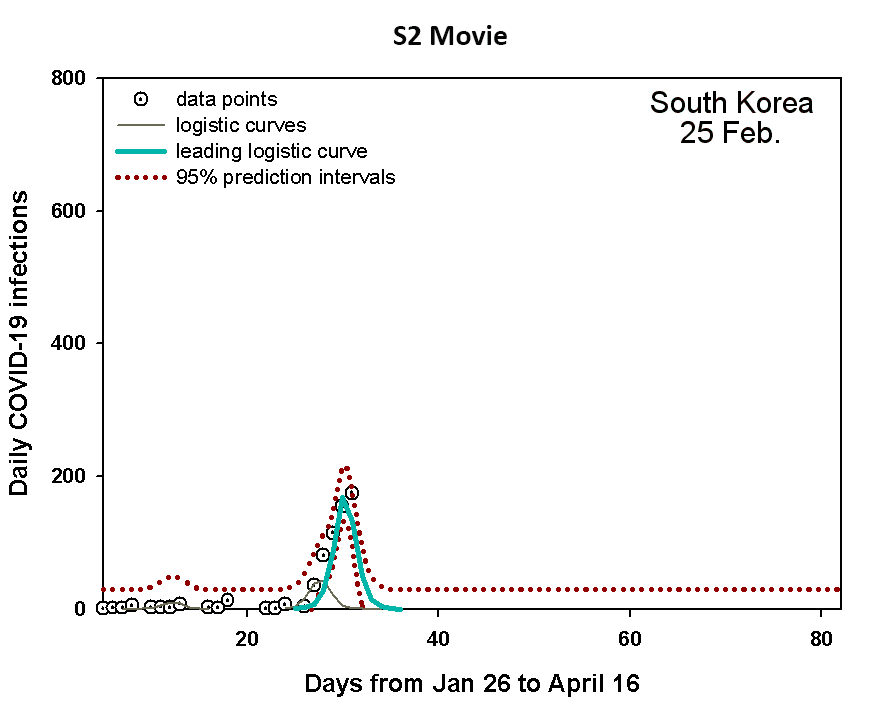

Supplement: S2 Movie — (GIF) [file pone.0250699.s003.gif]

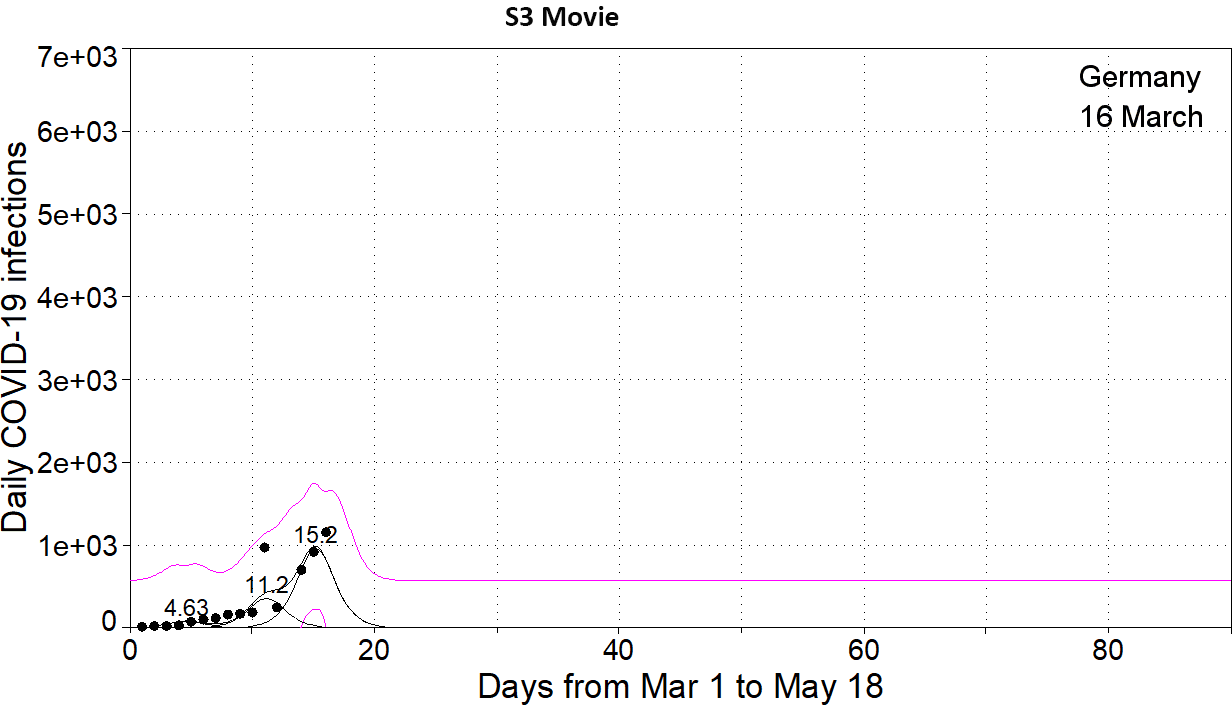

Supplement: S3 Movie — (GIF) [file pone.0250699.s004.gif]

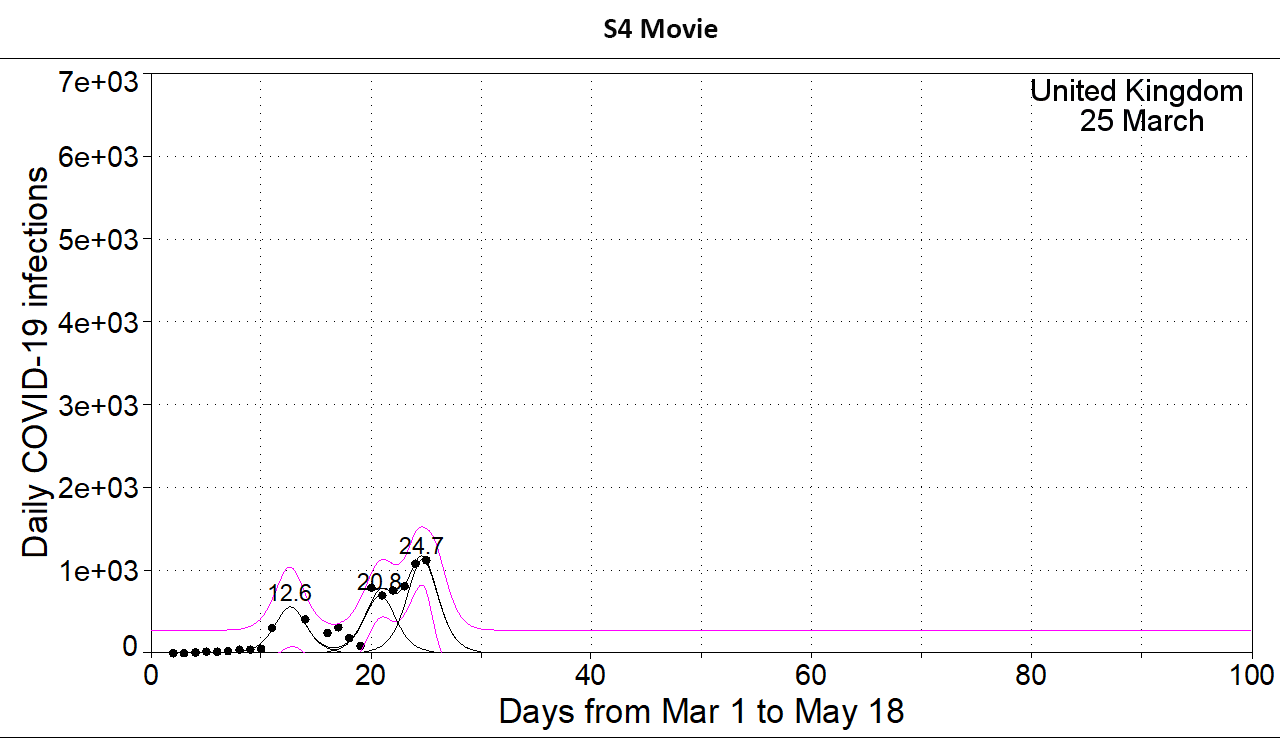

Supplement: S4 Movie — (GIF) [file pone.0250699.s005.gif]

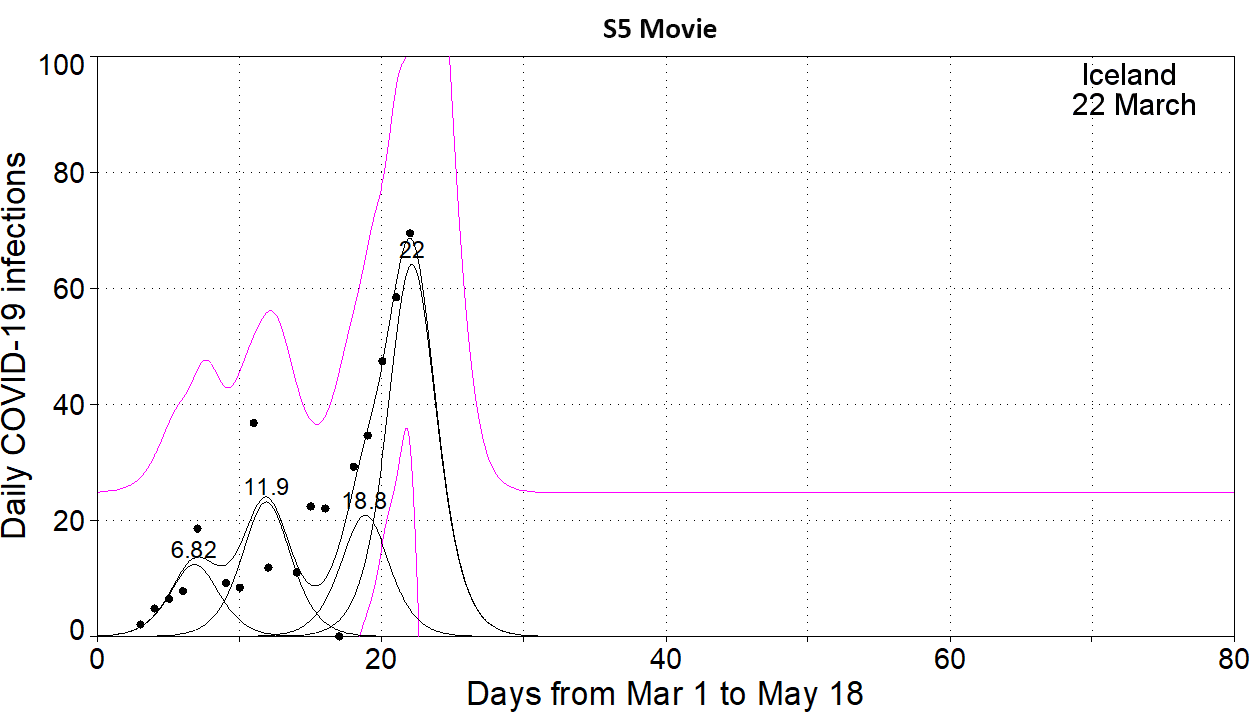

Supplement: S5 Movie — (GIF) [file pone.0250699.s006.gif]
